# Data literacy in genome research

**DOI:** 10.1515/jib-2023-0033

**Published:** 2023-12-05

**Authors:** Katharina Wolff, Ronja Friedhoff, Friderieke Schwarzer, Boas Pucker

**Affiliations:** Plant Biotechnology and Bioinformatics, Institute of Plant Biology & BRICS, TU Braunschweig, Braunschweig, Germany

**Keywords:** computational biology, genomics, sequencing, data literacy, bioinformatics, education

## Abstract

With an ever increasing amount of research data available, it becomes constantly more important to possess data literacy skills to benefit from this valuable resource. An integrative course was developed to teach students the fundamentals of data literacy through an engaging genome sequencing project. Each cohort of students performed planning of the experiment, DNA extraction, nanopore sequencing, genome sequence assembly, prediction of genes in the assembled sequence, and assignment of functional annotation terms to predicted genes. Students learned how to communicate science through writing a protocol in the form of a scientific paper, providing comments during a peer-review process, and presenting their findings as part of an international symposium. Many students enjoyed the opportunity to own a project and to work towards a meaningful objective.

## 1 Introduction

We live in a world of data with collections and databases growing in size and complexity at an ever increasing pace. Examples are the sequence databases European Nucleotide Archive [1], GenBank [2], SwissProt [3], and NGDC [4] that all showed an exponential growth in recent years [5]. Genomics is a research field that deals with inherently large data sets. Many researchers in the field are committed to open data and are sharing the sequencing data, genome sequences, and related data sets through well established databases. Examples of important plant genome databases are Phytozome [6], PLAZA [7], banana genome hub [8], Sol Genomics Network [9], and the rice genome hub [10]. While databases like ENA and GenBank are universal, other databases like the banana or rice genome hub support specific communities through focus on a set of closely related species. This free exchange of data allows large scale studies and provides the basis for benchmarking studies [5, 11]. Sequencing of genomes has produced ‘big data’ for over 20 years [12–14] and there are plans for future activities to systematically study the genomes across the full taxonomic diversity of plants [15, 16]. While initial genome sequencing projects were conducted by large international consortia [17], nowadays individual research groups can complete genome sequencing projects [16]. The onset of nanopore sequencing resulted in a democratization of sequencing that enables even students to engage in genomics.

Large data sets pose a valuable resource that could be harnessed for scientific discoveries, economic endeavors, and the good of society. Examples are genome sequences of crop wild relatives that can be utilized to identify pathogen resistance genes for introduction into high yield cultivars through breeding [18, 19]. However, a lack of knowledge about the methods for exploring data sets and the interpretation of data is an obstacle to gaining the aforementioned benefits. There are also some challenges concerning the access to sensitive data types e.g. in biomedical research [20]. Education about data management and data literacy is largely restricted to dedicated data science study programmes. However, we see a necessity to equip students of other subjects and especially in the life sciences with the necessary skills. This need was also recently summarized in the form of grand challenges in bioinformatics education [21]. Furthermore, it is important to teach data literacy and data management skills in close connection to the scientific discipline and not as an isolated subject. The combined teaching in bioinformatics and molecular biology [22] is an example for successful interdisciplinary education. Previous publications also reported success with transdisciplinary approaches used for diverse cohorts [23, 24] and the particular importance of a practical methodology and problem-based learning approaches [25].

Given the availability of data sets and the accessibility of cutting-edge sequencing technologies, we developed a data literacy course with a focus on genomics. Owing to our background in plant biology, we focus on plant genomics, but the concepts and methods are also applicable to research projects investigating other organisms.

## 2 Course content

**Figure 1:**
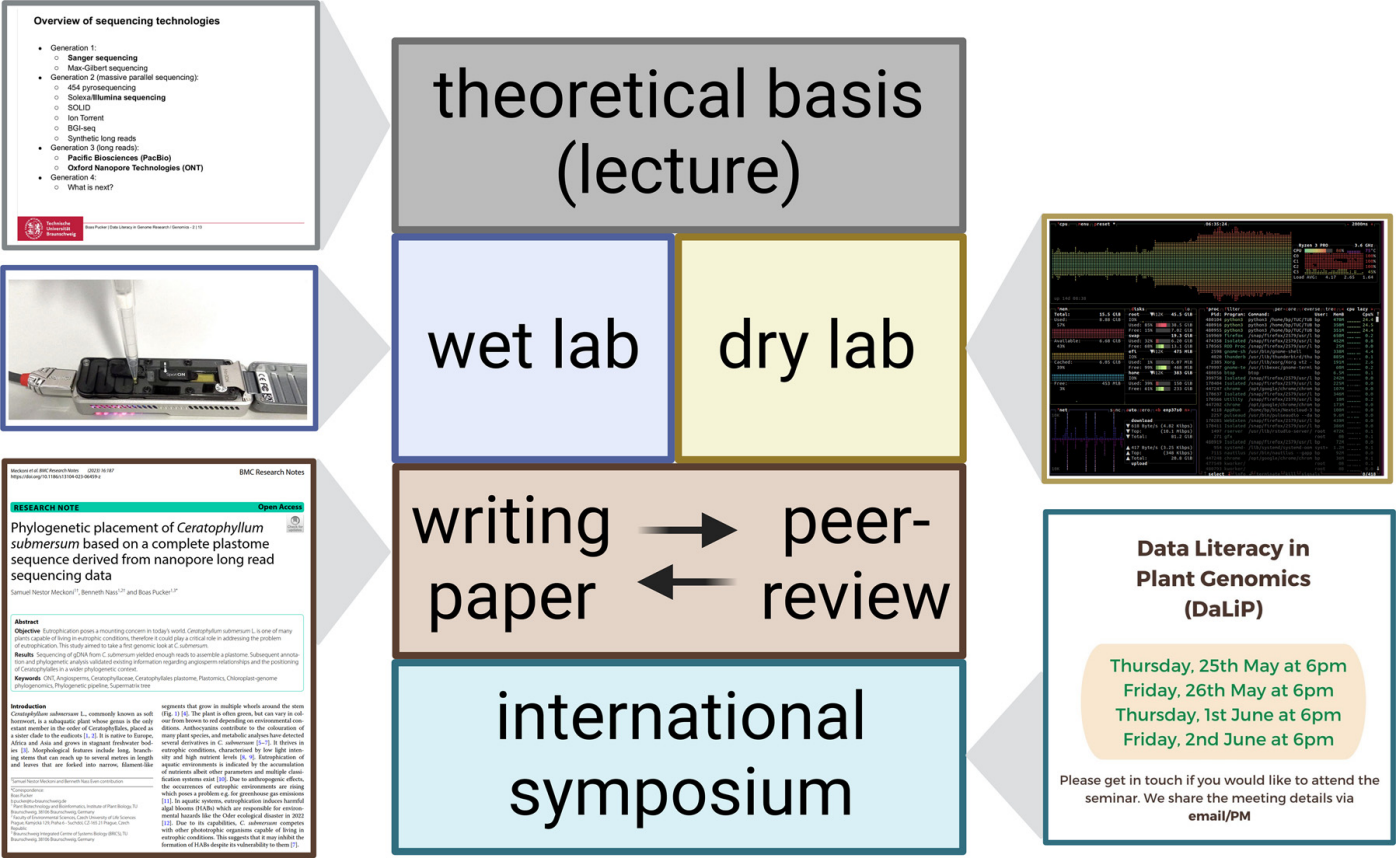
Overall structure of the module comprising lecture, practical parts, paper writing, and oral presentation of the results. Credits for taking a MinION picture and design of the symposium announcement to Melina Nowak. The screenshot belongs to a related open access publication [27].

The objective of this six-week course was to provide students with theoretical knowledge and hands-on experience of an entire genome sequencing project from the planning of the project to the reporting of final results (Figure 1). Theoretical background about genome sequencing strategies, sequencing technologies, and data analysis methods was provided through a lecture in the first week. Following open education principles, the materials of the lecture are freely available through GitHub [26]. The lecture enables the students to plan their own sequencing project. The precise objectives of the different cohorts differed and were subject to availability of plant material and students’ preferences. Courses running in the winter were relying on plants that were grown specifically for this purpose. Many cohorts investigated plant pigmentation of some kind, because a visible phenotype is advantageous in a teaching setting.

**Figure 2:**
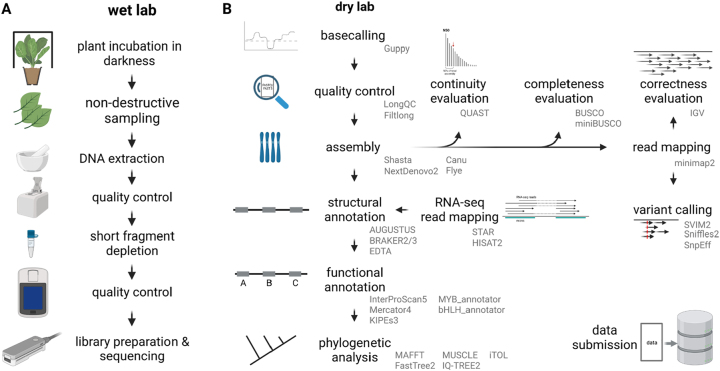
Graphical summary of practical course content. (A) Steps of the sequencing workflow performed in the wet lab. (B) Data analysis steps performed with bioinformatics tools. Names of selected tools are given for the different data analysis and processing steps.

The practical course part can be separated into two weeks of wet lab work and three weeks of data analysis (Figure 2). The students estimated the genome size by integrating public information about the species and closely related species. Next, the amount of required sequencing data was calculated and served as the basis for the planning of the sequencing experiment. DNA extraction was usually performed based on a universal CTAB protocol [28], but students had the possibility to explore other options. A NanoDrop measurement served as the first quality check. Only DNA containing samples were taken forward to a quality assessment via agarose gel. Students were able to roughly evaluate the fragment size and to determine the amount of RNA contamination on the gel. A precise DNA quantification was performed via Qubit measurement with a dsDNA broad range kit. The whole DNA extraction process was repeated several times to allow students to gain some routine and to see if the quality of the outcome improves.

DNA samples passing through all these iterative filters were subjected to removal of short DNA fragments with the Short Read Eliminator kit (Circulomics/PacBio). During the process students learned to make decisions about the next steps based on quality check results. Finally, students picked samples that were suitable for library preparation and nanopore sequencing. Students were provided with the SQK-LSK109 and SQK-LSK110 protocols for library preparation. Another Qubit measurement was performed prior to loading flow cells to ensure that the process was completed successfully. Students prepared R9.4.1 flow cells for sequencing and loaded their successfully prepared libraries. Sequencing was performed for about 16 h in most cases. The course was designed in a way that multiple students share a flow cell. One student started a sequencing run on a flow cell on the first day. The next student performed a flow cell washing on the next day and prepared and loaded a new library on the flow cell. Following this strategy, up to four students were able to use one flow cell.

During the first cohorts, the conversion of raw nanopore sequencing data into actual sequences (basecalling) was performed with Guppy (ONT) using a graphic card in the de.NBI cloud. After upgrading the local computational resources, real time basecalling was performed to allow students the monitoring of even more parameters during the sequencing experiments. Next, students assessed the quality of the generated data sets. LongQC [29] and Filtlong [30] were often applied for this step. Afterwards, the reads were subjected to a genome sequence assembly with different tools. Students were encouraged to identify suitable tools for this step. Frequently, Shasta [31] was deployed due to its short run time and low computational costs. Cohorts working on plant species with smaller genomes also tried other assemblers like Canu [32], NextDenovo2 [33], or Flye [34]. Different tools were deployed to evaluate the quality of the resulting assemblies with respect to the three ‘C’s: continuity, completeness, and correctness. While some cohorts relied on basic Python scripts for counting contig numbers and N50 calculation (contig_stats3.py, [35]), others applied QUAST [36]. BUSCO [37, 38] and compleasm [39] were frequent choices for the completeness assessment. Individual students tried to evaluate the assembly correctness through coverage analyses based on long read mapping with minimap2 [40] and alignment inspection with Integrative Genomics Viewer (IGV) [41, 42].

While the assembly of plant genome sequences is transitioning into a routine task, the structural and in particular the functional annotation are becoming the new bottlenecks [16]. Assembled genome sequences were structurally annotated with AUGUSTUS [43, 44], BRAKER2/3 [45, 46], or EDTA [47]. Since AUGUSTUS and BRAKER focus on protein coding genes, EDTA was applied to annotate transposable elements that comprise a substantial amount of the genome and consequently account for a substantial amount of the genome sequence. AUGUSTUS can perform a gene prediction without the integration of additional hints. BRAKER is based on AUGUSTUS and permits the use of RNA-seq data sets or sequences of a close relative as gene prediction hints. The mappings of RNA-seq reads for the generation of hints for the gene prediction process were performed with STAR [48, 49] or HISAT2 [50]. Students had the opportunity to explore other gene prediction tools as well.

The functional annotation was largely based on the identification of orthologs in *Arabidopsis thaliana* and a following transfer of the TAIR annotation terms. BLASTp [51, 52] was applied for the identification of reciprocal best BLAST hits (RBHs) which can serve as indicators for ortholog connections [35]. A general annotation of all predicted polypeptide sequences was also performed based on InterProScan5 [53] and Mercator4 [54, 55]. Additional annotation steps were performed based on the specific research question in the respective cohort. Projects focussing on flower pigmentation performed an in-depth annotation of the flavonoid biosynthesis via KIPEs [56]. Additionally, students analyzed the MYB and bHLH transcription factor families, which harbor important transcriptional regulators of the flavonoid biosynthesis, with dedicated tools [57, 58].

The relationship of individual candidate sequences was studied based on phylogenetic trees constructed by FastTree v2 [59] or IQ-TREE v2 [60] usually based on MAFFT v7 [61] or MUSCLE v5 [62] alignments. A customized Python script algntrim.py [63] or pxclsq [64] were applied for the alignment trimming i.e. to remove columns with low occupancy. Finally, the phylogenetic trees were visualized in iTOL [65]. Students learned how to root a tree and how to color different elements to highlight specific aspects.

An optional element in several courses was the detection of sequence variants based on a mapping of reads against a reference genome sequence. Depending on the particular research question in the respective course, this element was included. Students were encouraged to identify tools for the mapping of long reads. A frequently selected tool was minimap2 [40]. Conversion of SAM to BAM file format and other BAM processing steps were conducted with samtools [66]. SVIM2 [67] and Sniffles2 [68] were applied for the identification of sequence variants between the long reads and the reference genome sequence. The functional consequences of the detected sequence variants were predicted with SnpEff [69].

Students wrote their report about the entire course content in the form of a scientific paper. Background about the respective project and the fundamental research question were presented in the introduction. All methods applied by the students were presented in the method section. With respect to reproducibility, students were encouraged to include all necessary details like tool version number and parameters used in their analyses. This method section covers the wet lab and dry lab part of the course. The results section showcases the sequencing output and the findings of the following data analysis. The discussion section allowed the students to demonstrate their newly acquired skills by interpreting their large data sets. This requires an integration of various analysis results to answer a biological question. A peer-review process was exercised to assess and further improve the report quality and give the students a first hand experience of the steps involved in the publishing process. If project planning, conduction of all experiments, and data analysis were very successful, students of sequencing courses could submit their results for publication [27].

An online symposium with an international audience concluded the practical courses. This was the opportunity for students to demonstrate their ability to present their main findings in a concise presentation and to defend their choice of methods in the following discussion. All students of a cohort worked on the same biological question thus their reports would only differ in the methodological approaches they applied. To avoid strong redundancy throughout an international symposium, participants of different cohorts were mixed.

## 3 Teaching techniques and lessons learned

**Figure 3:**
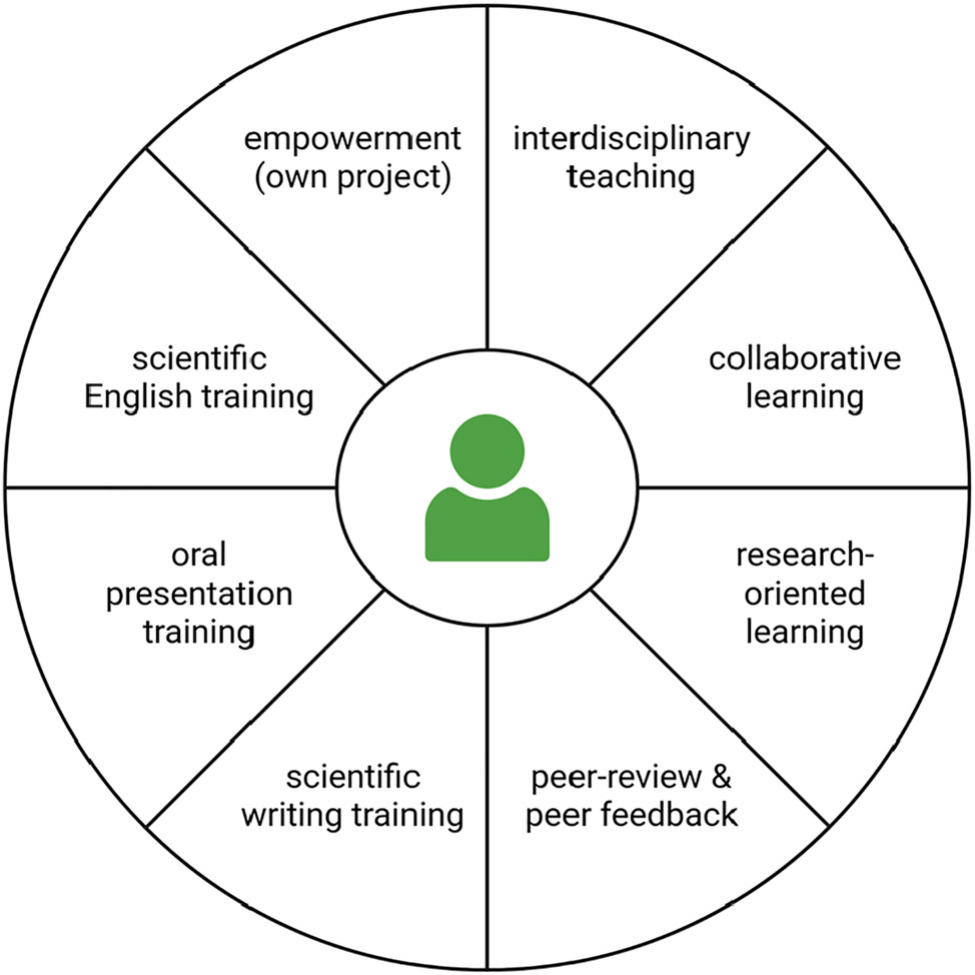
Summary of the didactic elements embedded in the course. (1) Interdisciplinary teaching allows the students to connect elements of different subjects and think about a specific project without borders imposed by different subjects. (2) Collaborative learning has many advantages, one of which is an increased motivation. (3) Research-oriented learning makes it obvious to students why they need to acquire certain skills, which again boosts motivation. It provides also a solid basis for upcoming challenges in a research career. (4) Utilizing peer-review and peer-feedback are successful teaching methods, because students can benefit from providing and from receiving comments. We utilized these methods throughout the course at various stages. (5), (6), and (7) Were all connected to the final presentation of the project in the form of a scientific publication and an oral presentation during an international symposium. (8) Empowering students by allowing them to develop their own project resulted in a motivation boost that we consider crucial for the success of this course.

We aimed for high motivation of the students and problem-oriented learning to ensure an optimal learning outcome. This course benefited from numerous teaching techniques that were previously tested, evaluated, and established with addition of ideas that were developed and evaluated over six cohorts of this course (Figure 3). An interdisciplinary project allows the students to connect their biology skills and knowledge with competencies in bioinformatics as this has proven successful before [22]. Students can participate in this course without comprehensive knowledge about various laboratory methods, because the project allows for an individual learning pace due to small group sizes. There are also several time slots within some protocols (e.g. 30 min centrifugation) that can be utilized to practice skills for the following steps or to go over theoretical concepts of complicated steps. A solid understanding of the wet lab and dry lab part of a project enables the students to communicate effectively with both molecular biologists and bioinformaticians. There are also synergistic effects as students improve in both fields. Although all students performed all tasks on their own to get hands-on experience, there were plenty of opportunities for exchange and collaborative learning. Writing a protocol about the entire course in the form of a scientific research article allows the students to apply all their newly acquired skills. The result was assessed and criticized by peers. This provides the students with suggestions for improvements and also trains their ability to provide constructive comments. Peer-reviews have been successfully used as teaching methods before [70]. Finally, students practiced presentation and communication skills when sharing their work and derived insights with an international audience as part of an online symposium. The students learned how to give a scientific presentation and how to interact with other scientists.

**Figure 4:**
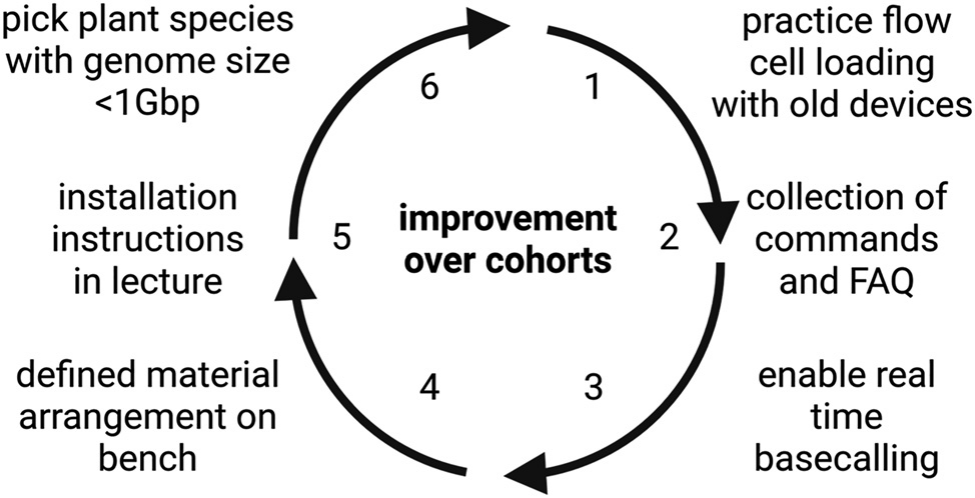
Continuous improvement of the Data Literacy in Genome Research course based on feedback and experience from six consecutive cohorts. (1) Extensively practicing the flow cell loading with old devices avoided the destruction of expensive materials, because the introduction of a single air bubble can already destroy a flow cell. (2) A collection of all fundamental commands enabled all students to start working in a Linux environment within the virtual machine more efficiently. A collection of the most frequently appearing errors and questions (FAQ) reduced the need to answer these questions redundantly. (3) Upgrading the computational resources to enable real time basecalling provided the students with additional monitoring options during the sequencing. (4) Defining the arrangement of different reagents and equipment on the bench reduced the chances of students dropping those expensive items. (5) Extending the introduction about software installation reduced frustration when students were facing too complex challenges. (6) Restricting the investigation of plants to genome sizes <1 Gbp helped to avoid too long waiting times when tools are running.

The described course was offered six times at TU Braunschweig during the period of one year with a total of up to 12 slots available per cohort. After participation in the course, students were asked to provide feedback for further improvements and to highlight strengths of the concept. Here we summarize their comments and derive some recommendations for future courses. Improvements were made between cohorts according to the feedback that students provided after participating in the course. While this ensures an ideal teaching outcome, it prevents a quantitative evaluation. It is also important that each cohort might have different and sometimes conflicting preferences concerning specific aspects. Nevertheless, some overarching points for improvement were identified through observation during the course and evaluation meetings with each cohort after completing the course (Figure 4).

While students appreciated the opportunity to pick their own tools for bioinformatic analyses, the installation of these tools was a substantial challenge for life scientists who had no prior Linux experience. Therefore, a collection of the most important commands was prepared and shared with students (Supplementary File 1). Spontaneous short presentations given by students and discussions were encouraged to address one of the big obstacles to successful bioinformatics research: installation of novel tools. In response to student requests, an introduction into software installation was included in the lecture. This includes an extension of the section about sharing tools to specifically introduce the general structure of a GitHub repository. Virtual environments are very important for the contained installation of new tools. Therefore, additional background information about configuring virtual environments was also added to the lecture. The practical part was extended by a short hands-on session about the installation of selected bioinformatics tools to demonstrate frequently used installation strategies. Following another student request, we updated the lecture to cover additional tools. One standard tool was defined for each analysis and students were asked to start their analysis with this tool before looking for alternatives. Nevertheless, we preserve the opportunity that students can identify their own tools and we encourage the students to do so. To ensure that students do not get lost in details, they were encouraged to construct a graphical overview to indicate how every tool contributes to the entire workflow.

Many learning successes are the consequence of errors. Therefore, we compiled a collection of frequently asked questions and frequently appearing errors (Supplementary File 1) which helps students to also learn from the errors of others. One example is that students are asked to prepare a document containing all their commands. This needs to be a plain text file (TXT) and not a Word document (DOCX). Learning about these tiny details can help students to avoid frustration.

To cover one additional aspect of data literacy, we included some strategies about literature search in the lecture, e.g. important databases and search strategies. Students can put their theoretical knowledge to practice by searching for publications pertinent to the specific project of the cohort. There is allocated time during the practical course part to ensure that all students gain sufficient experience with literature search, which was appreciated by the students. Experiencing success with small challenges on a daily basis enables students to feel confident in future analyses and has substantial advantages over a predefined set of commands that all students have to execute. Students liked the opportunity to explore their own ideas, to test alternative tools, and to follow up on their own research questions. As this does not require any additional preparations, we believe that this high degree of flexibility is transferable to many other courses.

Following a student request, we introduced short presentations by all participants about specific results that they obtained. Students were asked to comment on the results of others and also received feedback regarding their own work. These presentations summarized the content of each day at the end of the day and also at the beginning of the next day. In a similar way, the content of a week is summarized on Friday before leaving for the weekend. As repetition is an important element of learning, we expect to improve the long term learning outcome through these measures.

While the specific research question is different for each cohort, methods remain largely the same and allow the reuse of teaching materials. We consider different parts of the course as modules that can be combined in different ways. However, it is important to note that the precise run time of tools depends largely on the provided data set which can pose a challenge. In our experience, a genome size above 1 Gbp is not suitable for most analyses in the practical course due to an excessive runtime. Based on a script for the practical part and instructions for the bioinformatics section, the students are challenged to develop their own summarizing documentation (Supplementary File 2).

Students appreciated generating their own sequencing datasets and running analyses based on their own datasets. It created a feeling of responsibility and also achievement when completing the project. The motivational boost resulting from the continuous work on a specific dataset can help to increase the learning outcome. The analysis of complementary data sets by different groups of students was considered an excellent solution that makes the course even more exciting.

Close supervision of up to 12 students by two experienced plant scientists during the DNA extraction parts was described as excellent. The sequencing supervision was also perfect with one experienced scientist supervising up to 3 students at a time. The inclusivity and failure culture in the courses was rated as excellent by many students. Many students learned a lot by making mistakes. The speed and difficulty of the course were constantly adjusted to meet the requirements of the respective cohort. Many students recommended reducing the course capacity to 5–8 students per cohort. That would ensure a close supervision leading to optimal outcome. The calculation that four students would be able to share a flow cell was too optimistic for realistic course settings. While this would work in an ideal situation, some flow cells were destroyed by students, when they accidentally introduced air bubbles into the system. Despite all precautions, it is probably more realistic to assume that on average two students can share a flow cell.

Some students have already applied recently developed large language models (LMMs) like ChatGPT v3 (https://chat.openai.com/auth/login) or the Bing chat via Microsoft Edge (https://news.microsoft.com/the-new-Bing/). These artificial intelligence (AI) tools were rated as very helpful tools when searching for installation instructions. However, there are still cases where these LMMs fail to provide an accurate answer to a specific question. Future AI development will enable students to write their data analysis scripts with AI support and fundamentally change the way we need to teach coding and bioinformatics.

The proportion of one week with 3–4 h of lectures per day followed by two weeks of wet lab work and three weeks of bioinformatics was rated as very good by most students. Especially the combination of wet lab and dry lab work in the same course was appreciated. Many life science students have not experienced dry lab work before and would not have selected a course on the topic. The combination of wet lab and dry lab can be an elegant approach to expose such students to bioinformatics. Many students also liked the opportunity to experience all stages of the project from designing the experiments, performing the data generation and data analysis to the final presentation of their findings in the form of a written and oral report.

Sharing all course materials freely through GitHub [26] and other platforms is important for the students. This enables the students to access everything even after leaving TU Braunschweig which results in loss of access to all internal file exchange systems.

We evaluated different settings for the bioinformatic analysis part. Flipped classrooms and other concepts were tested, but students preferred to work on site in a seminar room. We consider this an important finding, because our expectation was that students would benefit from the increased flexibility and the saved time by not commuting to a university building. They explained that peer pressure is important for many of them to actually work on the project. The students suggested an alternative to presence in a seminar room: an online meeting where all students keep their video on.

## 4 Conclusions

This ‘Data Literacy in Genome Research’ course was developed as a teaching innovation connecting different subjects and enabling students to develop their own project. Over the course of six weeks, the students gained theoretical background knowledge and hands-on experiences in genomics and computational biology. We identified a number of key elements like the ‘own project’, peer-feedback, and opportunity for individual approaches. Now, we are utilizing our experiences to establish a permanent course with similar content. In line with open education concepts, we make our teaching materials and data sets freely available to the community.

## Supplementary Material

Supplementary Material Details

Supplementary Material Details
