# Supplementary material for: Data literacy in genome research
Source: J Integr Bioinform. 2023 Dec 5;20(4):20230033. doi: 10.1515/jib-2023-0033 (PMC10777367; doi:10.1515/jib-2023-0033)
Supplement: Supplementary file 1 — Supplementary Material Details [file j_jib-2023-0033_suppl_001.docx]

Supplements

Supplementary File 1: Basic commands for working in a virtual machine on linux.

Supplementary File 2: Documentation of the entire workflow by a course participant.
