# Supplementary material for: Data literacy in genome research
Source: J Integr Bioinform. 2023 Dec 5;20(4):20230033. doi: 10.1515/jib-2023-0033 (PMC10777367; doi:10.1515/jib-2023-0033)
Supplement: Supplementary file 2 — Supplementary Material Details [file j_jib-2023-0033_suppl_002.zip › SupplementaryFile1_CommandLineBasics_v1.1.html]

## Denbi cloud/linux command line – basics

- **IMPORTANT!**  This file does ONLY contain generalised Ports (-p 12345) and IP adresses (ubuntu@123.456.78.90) please replace these with the correct numbers.
- Generally, you will always either give the full path to a file/directory or be in the same directory as the file you want to access.
- You should always prewrite your commands in a text editor file; this also allows for easy documentation.
- Include the date in the folder/file name as it makes it easier to find later on.
- Never use spaces; instead, use underscores (\_) and include program-related or descriptive names.
- Use the middle mouse button to copy into the terminal.

### Connect to cloud

- Open the terminal.
- ssh -i /path/to/private/key ubuntu@123.456.78.90 -p 12345

### Change the directory folder

- General command:  cd NAME\_OF\_FOLDER
- /vol describes our base node and is the only folder that requires a "/" in front of it.
- /vol/data/ is our working volume.
- For example: cd /vol/data

### Display folder content

- ls → Stands for "list".
- ls -l → shows folder content and includes permissions and file size.
- ls -lh → shows the same as ls -l, but makes the file sizes easier to read.
- ls -a → shows hidden files as well.

### Display available storage space and file size

- df -h → shows all available storage volumes along with their storage capacity and current usage.
- du --max-depth=1 → shows the content and size of one folder structure, including one level below your current level.

### Make a new directory

- General command:  mkdir NAME\_OF\_FOLDER
- For example:  mkdir 20230628\_augustus\_output
- Include the **date**  in the folder name to make it easier to find later on. Never use spaces; instead, use **underscores**  (\_) and include program-related or descriptive names.

### Viewing text files in the cloud

- less FILENAME.txt  → loads the first few lines of the file, allows you to scroll through it; exit this mode by pressing **q**
- cat FILENAME.txt  → prints the entire document onto the terminal; only recommended for short files not entire for example fasta files
- head FILENAME.txt  → prints the first few lines onto the terminal; good to check for example the structure of a fasta header or table
- tail FILENAME.txt  → prints the last few lines of the document onto the terminal; good if you just want to check the current progress in a log file

### Deleting

- Deleting a file: rm FILENAME or rm /vol/data/path/to/file
- Deleting a directory: rm -r DIRECTORY\_NAME
- You need to be in the directory where the directory is located.
- /vol/data/member/data\_output is supposed to be deleted:
- cd /vol/data/member
- rm -r data\_output

### Data transfer between the denbi cloud and your computer

- Open a second terminal that is NOT connected to the cloud.
- General structure:   
  scp -P 12345 -i /path/to/private/key \   
  /path/to/file/you/want/to/transfer \   
  ubuntu@IP:/path/to/where/i/want/my/file/to/go/on/vm
- Transfer file from your computer to the cloud:
- scp -P 12345 -i /path/to/private/key \   
  /vol/cluster-data/Urtica/run.cfg \   
  ubuntu@123.456.78.90:/vol/data/members/Urtica/nextdenovo/
- Transfer file from the cloud to your computer:
- scp -P 12345 -i /path/to/private/key \   
  ubuntu@123.456.78.90:/vol/data/members/Urtica/nextdenovo/ \   
  /vol/cluster-data/Urtica/run.cfg
- The backslash (\) at the end of the line allows you to prewrite it in multiple lines.

### Copying and Moving Files Within the Denbi Cloud - Copy a file/directory: cp path/to/file/or/directory /path/to/duplicate/location/and/name - Move a file or folder: mv path/to/file/or/directory /path/to/new/location Different Installation Methods for Programs - Not all programs are available for every installation method. It's best to check the program documentation/readme/manual and follow the recommended installation method. - conda: conda install nameofcondapacket - It's also possible to create virtual environments: - conda create -n myenv → provides an environment so differently installed versions of, e.g., Python do not interfere with each other. - conda activate myenv → activates your environment. - Conda install NAMEOFPACKAGE → installs your desired program if it is available in the active environment. - pip: pip3 install NAMEOFPROGRAMM - git clone: git clone link/to/github/directory Check Running Programs and Terminate a Job - top/htop/btop → show general usage and all jobs running in different ways. - kill JOBID → terminates the job corresponding to the job ID you give. - nice → changes the priority of a job. Useful Tips and Tricks - You can automatically complete your directory path by pressing tab. - cd .. makes you go one directory backwards. - Commands: some programs require a long list of parameters and attributes which can be very confusing to type all in one line. Instead, use "\" at the end of one argument. - Adding & at the end of a command detaches it from the terminal and allows you to close the terminal/connection to the VM by typing “exit” without ending the running program. - It can be beneficial to store the terminal output in a separate file; there are several options to achieve that: - >20230515\_log\_nextpolish.txt → sends printout messages to the file. - 2>&1 → specifies that both standard output (STDOUT) and standard error (STDERR) are written into the file! CAUTION: some programs have the result as the standard output so there is a chance you mix error messages and your results into one file. - python2 /vol/cluster-data/Urtica/scripts/nextPolishWrapper.py \ --bam /vol/cluster-data/Urtica/20230512\_mapped\_assembly/mapping\_sorted.bam \ --seq /vol/cluster-data/Urtica/clean\_urtica\_assembly\_v01.fasta \ --out /vol/cluster-data/Urtica/20230515\_nextPolish \ --bam\_is\_sorted \ >20230515\_log\_nextpolish.txt 2>&1 & Different Programming Languages and File Endings - Script.jar → java script.jar - Script.py → python script.py (depending on the Python version: python2 script.py or python3 script.py) - Script.pl → perl script.pl FAQs - How do I find good tools for the task at hand? --> You can find tools by looking at publications dealing with a similar research question as yours, the usage of the different tools you can usually find on GitHub. - I downloaded a programm and want to know how to use it. Where can I find the information? --> You can either look up the README file on the GitHub repository or you start the tool and add --help. - I downloaded a script from github with wget but the file doesn't contain the code. --> Make sure to download the raw file from gihub or you end up with the html code of the website. You can access the raw file by opening the file on github and then pressing the RAW button. - I started a program but it stops with the error code: File not found. --> Check that there are no spelling errors in the path to the files, if you press TAB it autocompletes the path for you. - Error Code: python script.py not found --> Unless you are in the directory which contains the script.py file you need to give the full path to the script in order to start it - How do I stop a programm I started, when its not running in the background? --> Press CTRL and C - Is there any way to move a process to the background once I started it? --> Yes, you can press CTRL and Z once the programm stopped type bg into the terminal - How do I remove an entire folder? --> rm -r FOLDER - What does the Error "Permission denied" mean and how do I fix it? --> It means your programm tries to write into a writeprotected folder; either you change the permissions of the target folder with 'chmod' or you run the command with 'sudo', this gives you admin rights.
