# Supplementary material for: Data literacy in genome research
Source: J Integr Bioinform. 2023 Dec 5;20(4):20230033. doi: 10.1515/jib-2023-0033 (PMC10777367; doi:10.1515/jib-2023-0033)
Supplement: Supplementary file 2 — Supplementary Material Details [file j_jib-2023-0033_suppl_002.zip › SupplementaryFile2_Workflow_DaLit_Rieke.pdf]

Additional workflow steps in grey are methods, that would have been nessecary for other tools (read trimming for Flye assembler), that could have optimized our results (repeat masking) or further analyses (variant calling and read mapping). Unfortunately, we did not have time to perform them. Alternative tools for certain workflow steps are also indicated in grey.

Workflow Steps	Tools	Input	Output	Commands for Tools
**DNA-Extraction**	CTAB-based DNA extraction	Plant material	High molecular DNA	-
**Library Preparation & Sequencing**	Oxford Nanopore Technologies (SQK-LSK109, MinION)	High molecular DNA	Raw sequencing data in FAST5 format	-
**Basecalling**	Guppy, Bonito	Raw sequencing data in FAST5 format	Long reads in FASTQ format	guppy_basecaller<br>-i /path_to_fast5_directory<br>-s /path_to_ouput_directory<br>--compress_fastq<br>-x 'cuda:0'<br>--flowcell flow_cell_type<br>--kit sequencing_kit_type
**Quality Control**	Customized python script FASTQ_stats3_graphical_v0.2.py by Samuel Nestor Meckoni, https://codeberg.org/snmeckoni/scripts	Long reads in FASTQ format	Histogram of read length distribution	
**Read Trimming**	(read trimming was needed for Flye, but not for Shasta as read trimming is integrated)	Long reads in FASTQ format	Long reads in FASTQ format, filtered for >10 kb	
**Assembly**	Shasta, Canu, Flye	Long reads in FASTQ format	Contiqs in FASTA format	shasta-Linux-0.11.1<br>--input /fastq_file<br>--assemblyDirectory /output_directory<br>--config /config_file

**Quality Control**	BUSCO, stats, QUAST, NLR	Contiqs in FASTA format	Percentage of BUSCOs found	busco<br>--cpu number of cpu<br>--download_path /path_to_busco_database<br>-l /database_for_busco_search<br>-i /path_to_assembly_fasta<br>-o /output_directory<br>-m mode/sequence_type<br>-f (overwriting existing files)<br>>> /path_to_log_file<br>2>&1 & (errors into log file)
**Repeat Masking**	RepeatMasker			RepeatMasker<br>-e hmmer (engine)<br>-pa 5 (no. of processors)<br>-qq (rush job, less sensitive)<br>-species arabidopsis (nearest model sp.)<br>-no_is (no check for bacterial insertions)<br>-dir /output_directory<br>-small (type of masking)<br>/path_to_assembly_fasta<br>> /path_to_log_file &
**Struct. Annotation**	Augustus, GeMoMa, BRAKER3	Contiqs in FASTA format	mRNA, CDS, PEP sequences in FASTA and annotation GFF3 format	augustus<br>--gff3=on<br>--UTR=on<br>--uniqueGeneId=true<br>--protein=on<br>--cds=on<br>--species=arabidopsis<br>/path_to_assembly_fasta<br>> /path_to_output_gff3_file<br>2> /path_to_log_file &

**Funct. Annotation**	MYB annotator, KIPES, BLAST, InterProScan5, KEGG	Peptide sequences from structural annotation	Candidate sequences	python3 KIPEs3.py<br>--baits /path_to_baits_directory<br>--positions /path_to_residuals_directory<br>--out /output_directory<br>--subject /fasta_from_structural_annotation<br>--seqtype type_of_sequence<br><br>python3 MYB_annotator.py<br>--baits /path_to_baits_fasta<br>--info /path_to_baits_txt<br>--out /output_directory<br>--subject /fasta_from_structural_annotation<br>--fasttree /path_to_FastTree
**Variant Calling**				
**Read mapping**				
